# A CSF Background Suppression Scheme in Arterial Spin Labeling MRI

**DOI:** 10.1002/nbm.70191

**Published:** 2025-11-29

**Authors:** Zhiyi Hu, Wen Shi, Yifan Gou, Zihan Wang, Vivek S. Yedavalli, Doris D. Lin, Hanzhang Lu

**Affiliations:** ^1^ Department of Biomedical Engineering, School of Medicine Johns Hopkins University Baltimore Maryland USA; ^2^ The Russell H. Morgan Department of Radiology & Radiological Science, School of Medicine Johns Hopkins University Baltimore Maryland USA; ^3^ F. M. Kirby Research Center for Functional Brain Imaging Kennedy Krieger Research Institute Baltimore Maryland USA

**Keywords:** arterial spin labeling, arterial transit time, background suppression, cerebral blood flow, multi‐delay ASL

## Abstract

Arterial spin labeling (ASL) MRI suffers from low signal‐to‐noise ratio. Current background suppression (BS) methods focus on suppressing tissue signal. The present work aims to test the hypothesis that BS schemes to suppress CSF signal can produce a greater benefit, given the recent observations that water in CSF has more pulsation as part of the neurofluid circulation. We developed a CSF BS scheme that maximally suppresses residual CSF signal using two inversion pulses. Its performance was compared with regular BS and enhanced BS, both of which primarily suppress gray and white matter signals. All schemes were evaluated in single‐delay and multi‐delay pseudo‐continuous ASL (pCASL) MRI. The single‐delay scans assessed cerebral blood flow (CBF), while the multi‐delay scans measured both CBF and arterial transit time (ATT). Reproducibility was assessed using voxel‐wise coefficient of variation (CoV) and spatial Spearman correlation coefficients (Rs). When using the CSF BS scheme, CSF signals were suppressed to less than 1% of the equilibrium magnetization, with gray and white matter signals around 5% in opposite magnetization signs. Complex control/label subtraction effectively accounted for magnetization signs and obtained the correct difference signal. In single‐delay pCASL, CSF BS reduced visually apparent hyper‐ and hypo‐intensity signals in CBF maps, which were present in the tissue‐focused regular and enhanced BS schemes. These artifactual signal fluctuations were particularly pronounced in the brain‐stem regions where CSF pulsation was the most severe, but also spread along the z‐encoding direction. Quantitatively, CSF BS resulted in a lower voxel‐wise CoV (8.8%) and higher R_s_ (0.89) in the CBF maps, compared with tissue‐focused BS (CoV 18.8%, R_s_ 0.64). In multi‐delay pCASL, CSF BS yielded a CoV of 5.9% and 11.3% for CBF and ATT maps, respectively, outperforming both enhanced and regular BS. These results demonstrate that CSF BS can reduce spurious signals and improve image quality in ASL perfusion MRI.

## Graphical abstract

Arterial spin labeling MRI suffers from signal instability due to CSF pulsation, which are not fully addressed by conventional tissue‐focused background suppression (BS). We developed a CSF BS scheme and demonstrated its advantages in both single‐ and multi‐delay ASL. CSF BS reduced spurious ASL signal and improved reproducibility in both settings, yielding more reliable CBF and ATT maps than tissue‐focused BS approaches.

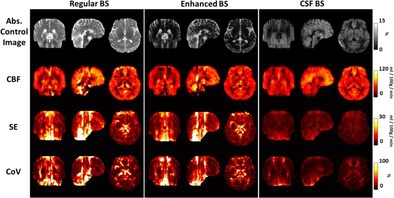

**Abbreviations**
ANOVA: analysis‐of‐variation
ASL: arterial spin labeling
ATT: arterial transit time
BS: background suppression
CBF: cerebral blood flow
CoV: coefficient‐of‐variation
FOCI: frequency offset corrected inversion
GRASE: gradient‐ and spin‐echo
pCASL: pseudo‐continuous arterial spin labeling
PLD: post‐labeling delay
Rs: Spearman correlation coefficients
SE: standard error
SNR: signal‐to‐noise ratio

## 1 Introductions

Arterial spin labeling (ASL) perfusion MRI is a noninvasive technique that allows quantitative mapping of cerebral blood flow (CBF) and related hemodynamic parameters without the use of contrast agents [1, 2]. ASL has found clinical applications such as in stroke [3, 4, 5], brain tumors [6, 7], hypoxic–ischemic encephalopathy [8, 9], and neurodegenerative diseases [10, 11, 12].

Despite these promises, a key limitation of ASL is its inherently low signal‐to‐noise ratio (SNR). ASL measures perfusion by magnetically labeling arterial blood water and detecting the perfusion signal through subtraction of the labeled image from a control image. However, arterial blood water replaces only ~1% of the water molecules in brain tissue each second, resulting in perfusion signals that are typically less than 1% of the equilibrium magnetization [2]. This small signal amplitude makes ASL particularly susceptible to noise, including signal fluctuations from static tissues that are not related to perfusion. To address this issue, background suppression (BS) techniques have been proposed to attenuate static tissue signals and suppress physiological fluctuations [13, 14]. The BS techniques employ nonselective inversion pulses to modulate the amplitude of the static spins while preserving the difference between the control and labeled images. Although BS has been extensively used in most ASL protocols and demonstrated an improvement in SNR [2, 14, 15], the existing schemes have two remaining limitations. One is that the static tissue has not been fully suppressed, due to the need to maintain sufficient magnetization to ensure that the MR signal is always positive (so that control signal > labeled signal). The second is that the current BS schemes have largely aimed toward suppressing gray and white matter signals [16, 17, 18, 19]. Recent new evidence on neurofluid circulation suggested that CSF pulsation is more pervasive than previously thought [20, 21, 22, 23]. Therefore, CSF signal in ASL may be a major source of physiological noise, and its suppression represents an opportunity to improve the SNR of ASL perfusion measurement [24].

In this study, we aim to develop improved BS strategies and examine their impact on ASL data quality. We first developed an enhanced BS scheme that is not limited by the signs of the magnetization by utilizing complex ASL data rather than the conventional magnitude data. This scheme allows us to suppress the static gray and white matter signal to be less than 1% of the equilibrium signal. We next expanded the scheme to focus on suppressing the CSF signal, as opposed to brain tissue, and assessed its benefits. The performance of these BS strategies was evaluated in both single‐delay and multi‐delay pseudo‐continuous ASL (pCASL).

## 2 Methods

### 2.1 BS Schemes in ASL

**FIGURE 1:**
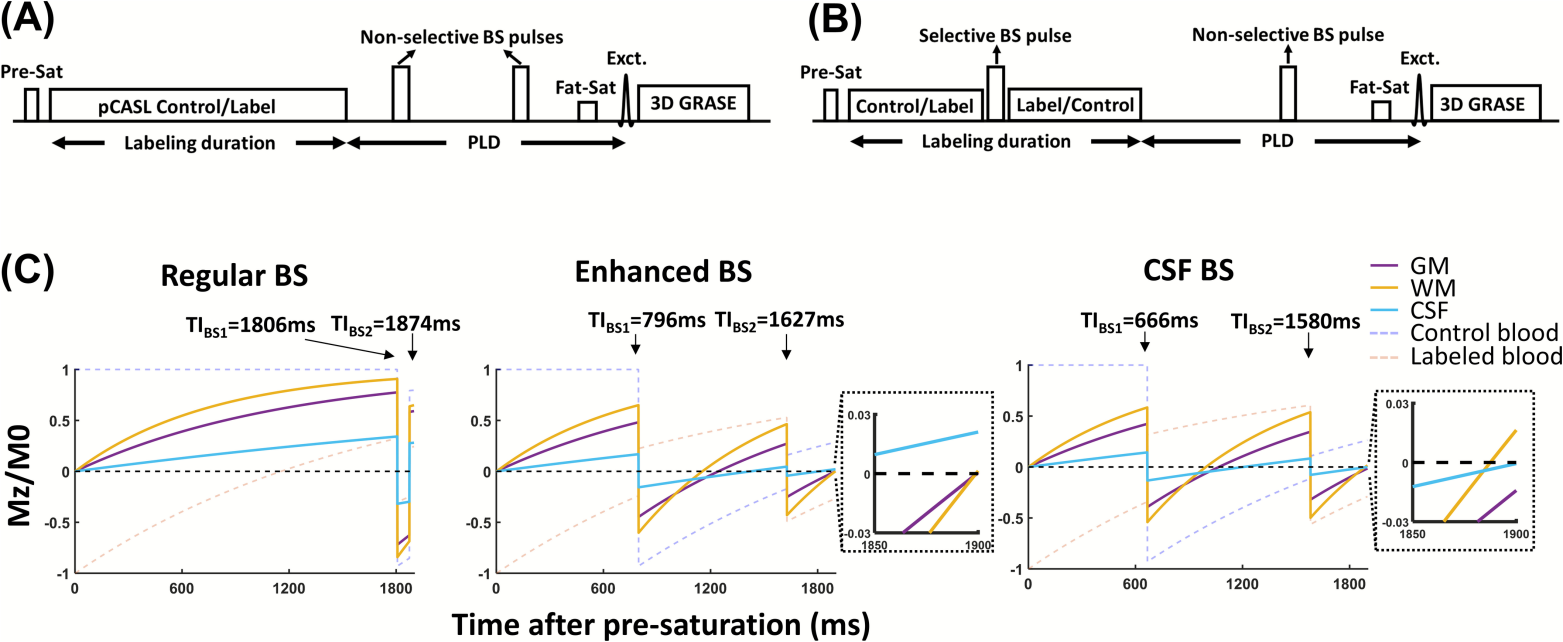
Illustration of pCASL MRI sequence with background suppression (BS). The sequence consists of a pre‐saturation immediately before the labeling module. Two BS inversion pulses are applied. (A) Constrained BS implementation, where the inversion pulses can only be placed during PLD. (B) Unconstrained BS implementation, where the inversion pulses can be placed during either the labeling period or the PLD. (C) Simulated longitudinal magnetization under different BS schemes, with insets showing a zoomed‐in view of the residual tissue signal at the time of excitation. pCASL parameters were labeling duration = 1800 ms, PLD = 100 ms. TI_BS1_ and TI_BS2_ denote the inversion time of the two BS pulses relative to the pre‐saturation module. Exct. = excitation, Fat‐Sat = fat saturation, GM = gray matter, Pre‐Sat = pre‐saturation, WM = white matter.

An ASL pulse sequence with a standard BS method is shown in Figure 1A. This BS method is referred to as constrained BS, where the BS RF pulses are only placed during the post‐labeling delay (PLD) period but not during the labeling period [16]. Another BS method, referred to as unconstrained BS, is shown in Figure 1B, in which the BS RF pulses can be placed during both PLD and labeling periods [16].

**FIGURE 2:**
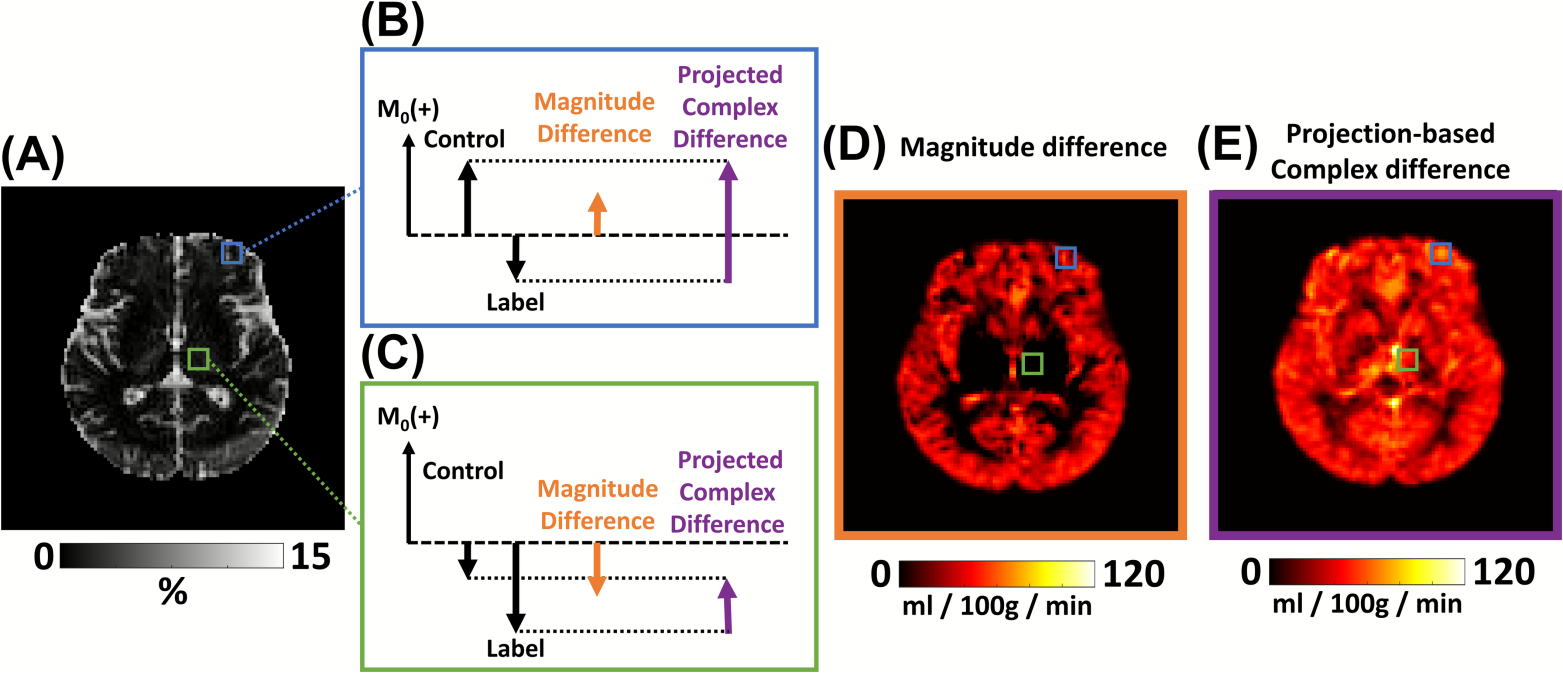
Illustration of the necessity of projection‐based complex subtraction for enhanced background suppression (enhanced BS). (A) Absolute control image with enhanced BS, expressed as a percentage of the M0 signal. (B) Illustration of a scenario where the control magnetization is positive and the label magnetization is negative. In this case, magnitude subtraction underestimates the ASL signal. (C) Illustration of a scenario where both control and label magnetizations are negative. In this case, magnitude subtraction produces an artifactual negative ASL signal. (D) CBF map obtained from magnitude subtraction (|control| − |label|), showing numerous voxels with low or negative values. (E) CBF map obtained from projection‐based complex subtraction, in which control and label complex vectors are projected onto the M0 direction to obtain signed scalars prior to subtraction. This approach corrects the erroneous CBF values observed with magnitude subtraction.

The present study compared three BS schemes: CSF BS, enhanced BS, and regular BS. The regular BS aligned with the practice employed in most manufacturers' product ASL sequences and used constrained BS. This scheme aimed to reduce static tissue signal to approximately 5% of its equilibrium magnetization [25, 26]. Notably, it ensures that the magnetization of a voxel is always along the positive direction; thus, one does not need to save complex data or estimate the sign of the MRI signal. The enhanced BS scheme employed unconstrained BS when needed and aimed to minimize residual signals in both gray and white matters regardless of the signs of the magnetizations [16, 19]. As a result, complex data are needed to ensure proper signal subtraction in ASL, as illustrated in Figure 2. CSF BS aimed to maximally suppress signals from the CSF at the expense of some residual gray and white matter signals. Complex data were also used in the CSF BS scheme.

All three BS schemes consisted of a pre‐saturation pulse applied immediately before the labeling period, followed by two inversion pulses prior to image acquisition (Figure 1A for regular BS and Figure 1B for enhanced and CSF BS). The difference between the schemes was the timing of the inversion pulses, with the additional constraint that, for the regular BS scheme, the inversion pulse cannot appear during the labeling period.

Simulations were performed to identify the optimal inversion pulse timings. The following parameter assumptions were used: T_1,gray matter_ = 1209 ms, T_1,white matter_ = 758 ms [27], T_1,CSF_ = 4308 ms [28], T_1,blood_ = 1664 ms [29]. The enhanced BS aimed to achieve gray and white matter signals to be less than 1% of equilibrium magnetization, with no specific constraints on the CSF signal intensity. The CSF BS aimed to achieve CSF signal to be less than 1% while minimizing both gray and white matter signals. Because of the gray and white matter T_1_ differences, this usually resulted in gray and white matter magnetizations having opposite polarities. Figure 1C displays representative simulations of longitudinal magnetizations when using these BS schemes, for a sequence with labeling duration = 1800 ms and a PLD = 100 ms.

### 2.2 General Experimental Methods

The study protocols were approved by the Institutional Review Board of the Johns Hopkins University. Written informed consent was obtained from each participant prior to the study procedures. All participants self‐identified as healthy and were confirmed to have no incidental abnormalities on anatomical scans reviewed by a board‐certified radiologist. MRI data were acquired on a 3‐T Siemens Prisma system (Siemens Healthineers, Erlangen, Germany) using a body coil for transmission and a 32‐channel head coil for receiving. This report consists of two experimental studies focusing on BS schemes in single‐delay and multi‐delay settings, respectively.

### 2.3 Study 1: Comparison of BS Schemes in Single‐Delay pCASL

Ten subjects (mean age = 26.8 ± 5.2 years; six females) were enrolled. Each subject received pCASL MRI under the CSF, enhanced, and regular BS schemes. Table 1 summarizes the timing of the BS pulses and the residual signals. Each BS scheme was assessed using two acquisition protocols: (1) single‐shot 3D gradient‐ and spin‐echo (GRASE), FOV = 220 × 220 × 144 mm^3^, voxel size = 3.4 × 3.4 × 4 mm^3^, TR/TE = 5520/36.12 ms, echo‐train length = 1444.8 ms, number of pairs = 20, scan time = 3.8 min; (2) segmented GRASE, FOV = 220 × 220 × 140.4 mm^3^, voxel size = 2.1 × 2.1 × 2.7 mm^3^, TR/TE = 5150/38.14 ms, 4 segments along the z‐direction, echo‐train length = 1067.9 ms, number of pairs = 12, scan time = 8.3 min. Other parameters were identical for both protocols and included labeling duration = 1800 ms, PLD = 2200 ms. An M0 image was also acquired for each protocol using a TR of 10 s.

**TABLE 1:** Inversion time and simulated residual signals of different BS schemes.

BS scheme	TI_BS1_ (ms)	TI_BS2_ (ms)	GM (%)	WM (%)	CSF (%)
Regular	2336	3585	5.2	7.9	12.5
Enhanced	2326	3630	−0.3	0.8	10.4
CSF	1806	3550	−7.0	2.9	−0.2

Abbreviations: BS = background suppression, GM = gray matter, TI = inversion time after pre‐saturation, WM = white matter.

Raw ASL data were processed offline using in‐house MATLAB (MathWorks, Natick, MA) scripts. MRI signals were initially stored as complex numbers. A representative image acquired with enhanced BS was shown in Figure 2A. For both CSF and enhanced BS schemes where the magnetization may be either positive or negative, the complex control and label vectors were projected onto the direction of the M0 signal vector [26] to obtain a signed scalar (Figure 2B,C). This allows the correct calculation of the control‐minus‐label difference for perfusion quantification (Figure 2E). Note this projection‐based approach differs from magnitude subtraction (|control| − |label|), which can underestimate or invert the ASL difference when control and label have opposite signs (Figure 2D), and from calculating the magnitude of the complex difference (|control − label|), which yields positively biased signal. For regular BS, control and label images were obtained by calculating the magnitude of the complex vectors, consistent with the standard ASL processing practice.

The ASL data were preprocessed using Statistical Parametric Mapping Version 12 (SPM12, University College London, London, UK). The control and label images were realigned to the first dynamic for motion correction. CBF maps were calculated based on a standard single‐compartment model [2] and smoothed using a Gaussian filter with a full width half maximum (FWHM) of 3 mm. Voxel‐wise temporal standard error (SE) and coefficient of variation (CoV) were computed to evaluate signal variability. The CoV was obtained by dividing SE by the mean signal. Spatial reproducibility was evaluated by dividing the ASL data into two halves, corresponding to 1 min 53 s per subset for single‐shot and 4 min 10s per subset for segmented acquisition. Spatial Spearman correlation coefficients (R_s_) were then calculated between the CBF maps. A Spearman correlation was used so that the coefficient is not sensitive to outlier voxels.

For statistical analysis, comparison among the BS schemes was performed using repeated‐measures analysis of variation (ANOVA), followed by Tukey's post hoc test. A *p* value less than 0.05 was considered statistically significant.

### 2.4 Study 2: Comparison of BS Schemes in Multi‐Delay pCASL

Eleven subjects (mean age = 27.1 ± 2.9 years; five females) were enrolled. The multi‐delay pCASL sequences used a segmented 3D GRASE acquisition with the following parameters: FOV = 220 × 220 × 144 mm^3^, voxel size = 3.4 × 3.4 × 4 mm^3^, TE = 36.14 ms, turbo‐factor = 10, echo‐train length = 361.4 ms, and labeling duration = 1800 ms. Five PLDs were used, namely, 100, 100, 1275, 1800, and 2100 ms, following an optimized protocol [30]. The shortest possible TR was used for each delay, resulting in variable TRs across PLDs (ranging from 2340 to 4340 ms). The total scan time for the multi‐delay ASL acquisition, including all PLDs, was 4.5 min. Each BS scheme was repeated once to allow the assessment of test–retest reproducibility. An M0 scan was also acquired.

The timings of the BS pulses and corresponding residual signals are summarized in Table S1. Note that, for inversion pulses placed during the PLD, nonselective hyperbolic secant pulses were used. For inversion pulses placed during the labeling period, slab‐selective frequency offset corrected inversion (FOCI) pulses were used to invert the spins distal to the labeling plane. The slab‐selective FOCI pulse ensures that the spins labeled before and after the pulse are additive rather than canceling each other out.

ASL image reconstruction and processing steps were similar to those described in Study 1. To obtain quantitative perfusion values from the multi‐delay data, arterial transit time (ATT) was estimated using a signal‐weighted delay method described by Dai et al. [31]. Following the ATT estimation, CBF was obtained based on an ASL kinetic model [32]. To assess the test–retest reliability, voxel‐wise CoV values were calculated for the CBF‐weighted images at each PLD as well as for the quantitative CBF and ATT maps using the repeated scans. R_s_ was also computed for the CBF and ATT maps.

Statistical analysis steps were similar to those described in Study 1. Bonferroni correction was performed to account for multiple comparisons for different PLDs.

## 3 Results

### 3.1 Study 1: Comparison of BS Schemes in Single‐Delay pCASL

**FIGURE 3:**
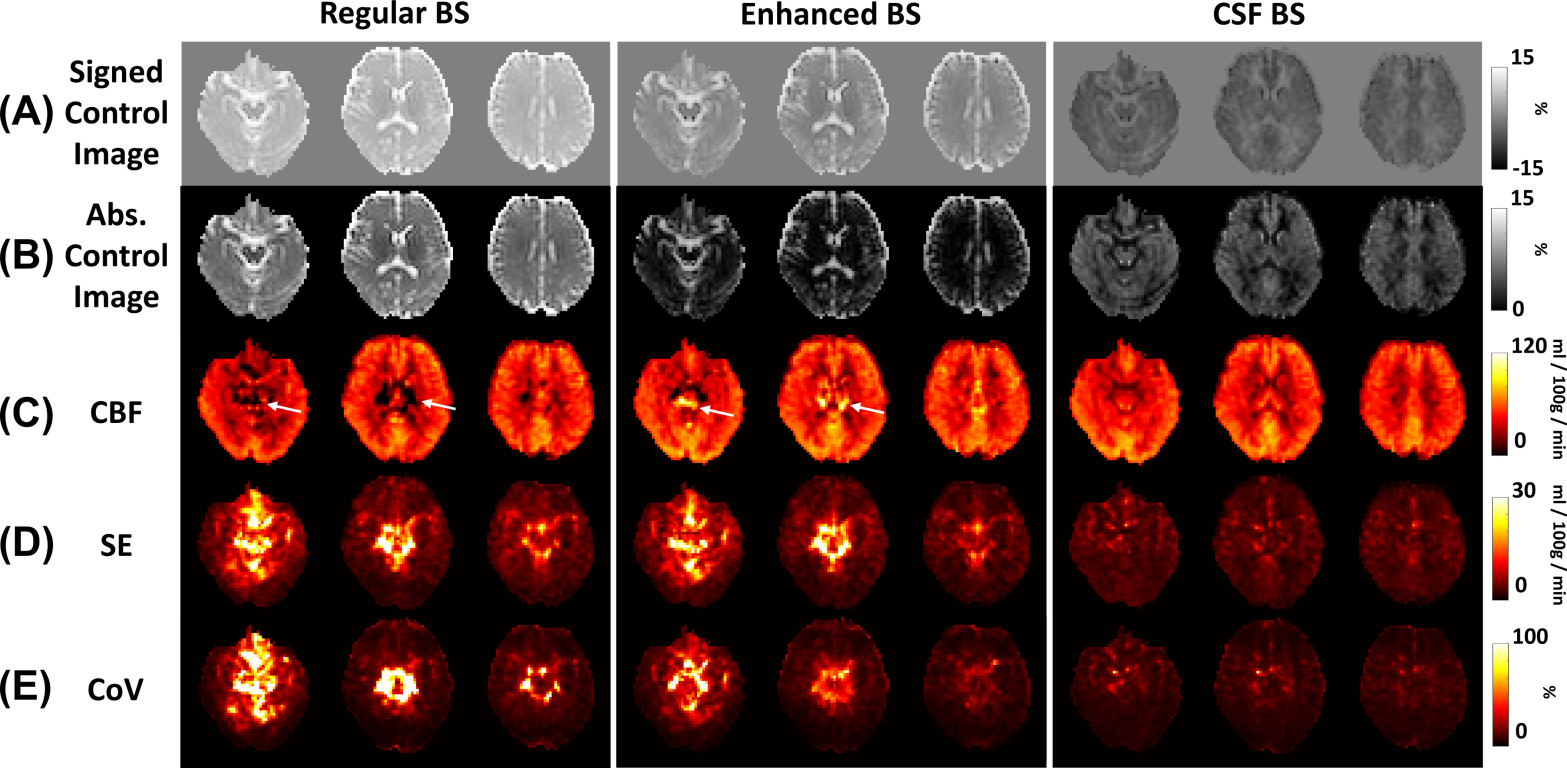
Images of different BS schemes using single‐shot 3D GRASE acquisition in a representative subject. (A) Signed control image, obtained by projecting control images onto the M0 images and expressed as a percentage of the M0 signal. (B) Absolute control image. (C) Cerebral blood flow (CBF) maps. Arrows indicate hypo‐ or hyper‐intense spurious signals due to CSF pulsation. (D) Temporal standard error (SE) maps across measurements. (E) Coefficient of variation (CoV) maps, showing high variability in regions rich in both large arteries and CSF.

**FIGURE 4:**
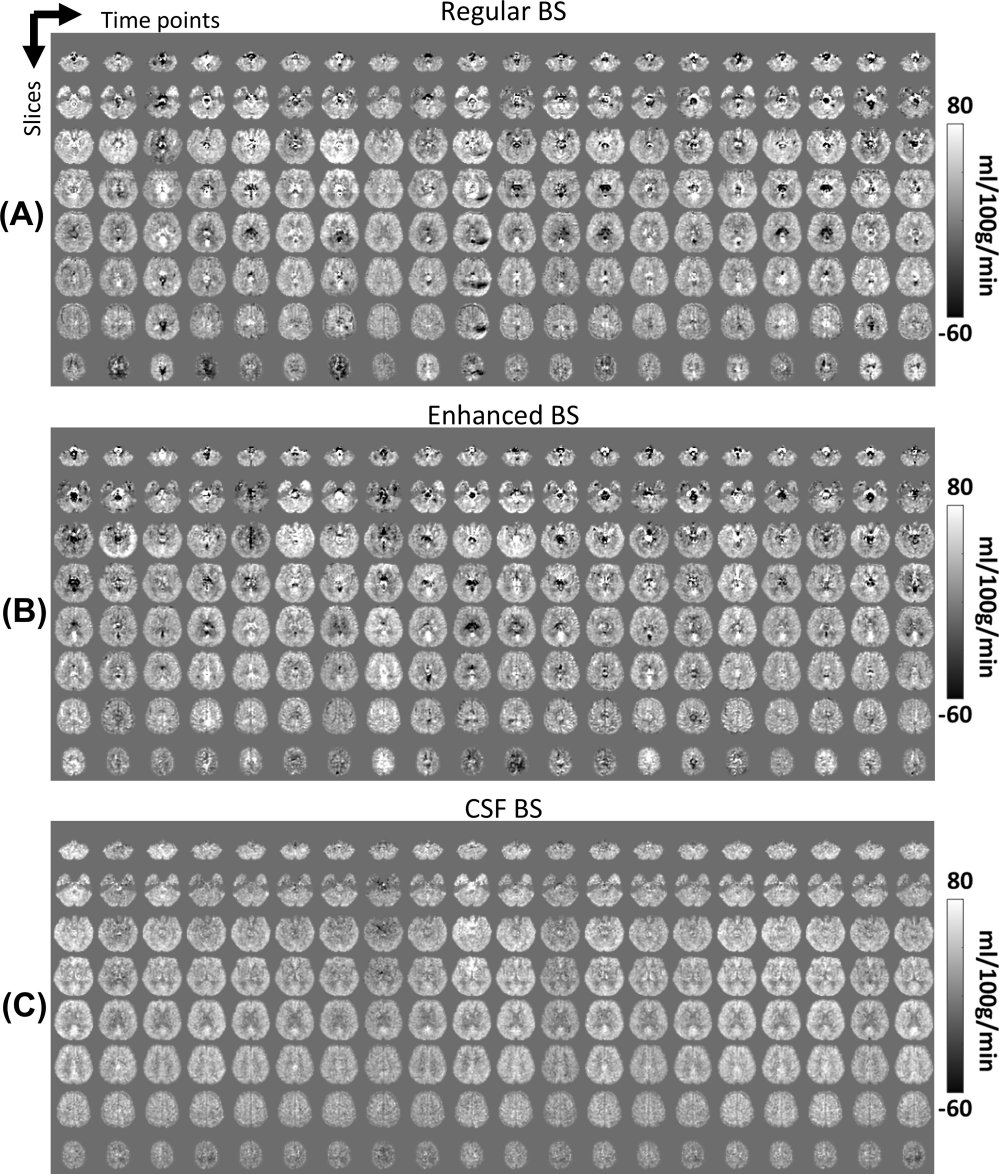
CBF maps from single‐pair control–label subtraction acquired with different background suppression (BS) schemes in a representative subject. Data were collected using single‐shot 3D GRASE. Each column shows one CBF volume from a single control–label pair (acquisition time = 11 s). (A) Regular BS. (B) Enhanced BS. (C) CSF BS.

Figure 3A,B shows single‐shot pCASL control images from a representative subject, illustrating the amount of residual signal under different BS schemes. It can be seen that the enhanced BS scheme minimized gray and white matter signals (< 1.5%), but the CSF signal is still relatively high (~10%). In contrast, the CSF BS scheme attenuated CSF signal to be negligible (< 1%) while containing some residual gray and white matter signals (~5%). Figure 3C displays the resulting CBF maps. Temporal SE and CoV maps are shown in Figure 3D,E. Visual inspection suggested that the image quality in regular and enhanced BS is similar. On the other hand, benefits of CSF BS can be seen in regions rich in both large arteries and CSF where regular and enhanced BS images revealed some hypo‐ and hyper‐intense artifacts (white arrows). Due to the 3D acquisition in GRASE, these effects are also spread along the z‐encoding direction to adjacent slices. Notably, CSF BS scheme revealed the least amounts of bright/dark artifacts and the lowest CoV. Additionally, we found that, using the CSF BS scheme, a single control‐and‐label pair (taking 11 s to acquire) was able to yield stable CBF images, as illustrated in Figure 4.

**FIGURE 5:**
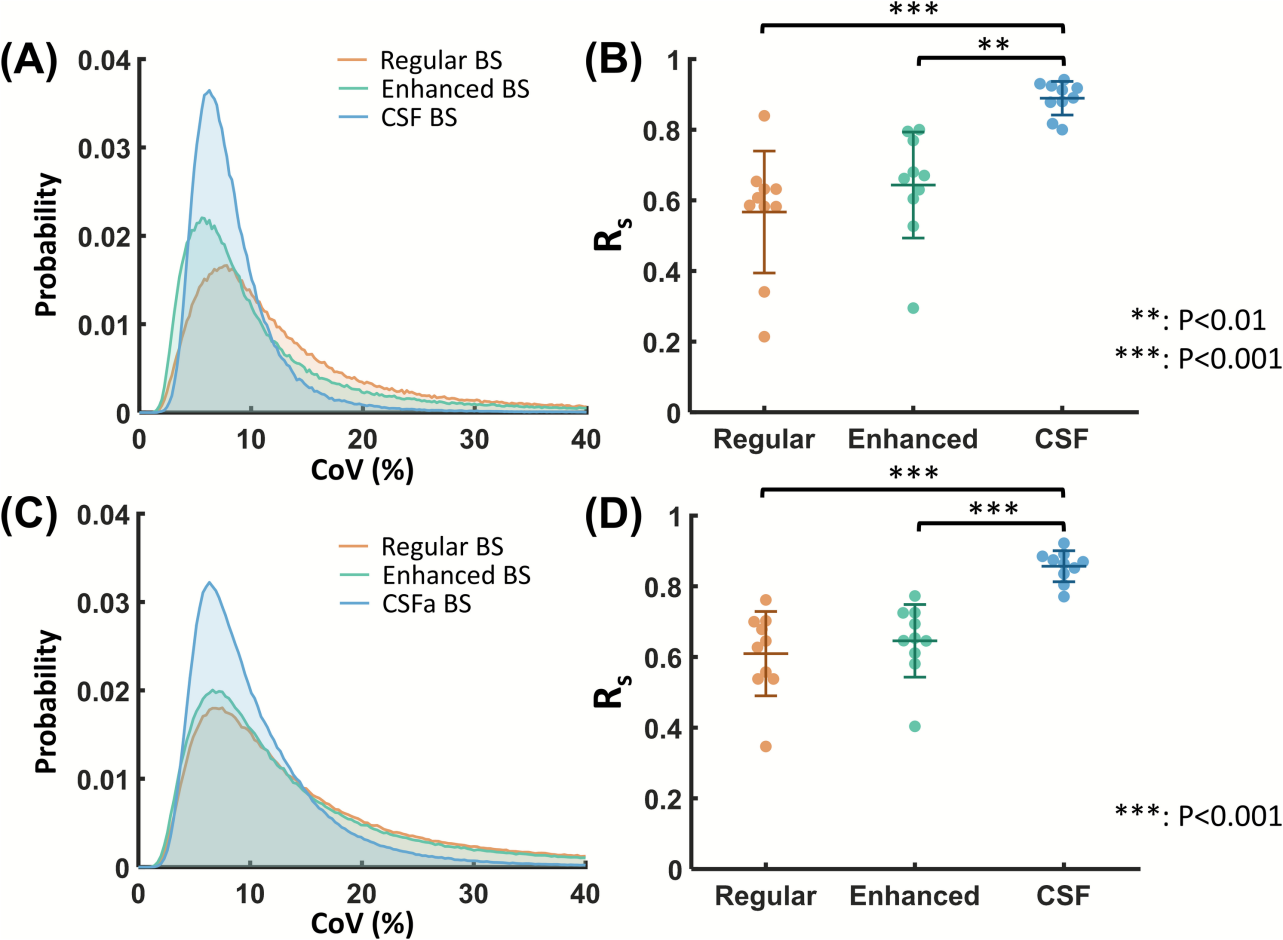
Comparison of data reproducibility across different BS schemes. (A) Group‐averaged normalized histograms of voxel‐wise coefficient‐of‐variation (CoV) values for single‐shot acquisition. (B) Spatial Spearman correlation coefficient (R_s_) between CBF maps for single‐shot acquisition. (C) Group‐averaged normalized histograms of voxel‐wise CoV values for segmented acquisition. (D) R_s_ between CBF maps for segmented acquisition. Error bars denote the standard deviation across subjects. The *p* values were obtained using Tukey's post hoc test following repeated‐measures ANOVA.

Quantitative results were also examined. No significant differences in whole‐brain CBF values were found among the three BS schemes (ANOVA, *p* = 0.42). Figure 5A presents group‐averaged normalized histograms of voxel‐wise CoV values. Compared to regular and enhanced BS, CSF BS exhibited a narrower distribution with a shorter tail, indicating fewer spurious voxels. Mean voxel‐wise CoV values were the lowest in CSF BS (8.8 ± 1.1%), followed by enhanced BS (18.8 ± 2.4%) and regular BS (22.1 ± 3.3%) (*p* < 0.001 for CSF BS vs. enhanced and regular BS). Voxel‐wise comparisons of CoV maps revealed that statistically lower CoV with CSF BS was mainly observed in CSF‐rich regions and surrounding regions affected by spatial smearing. There were no gray or white matter specific effects (Figure S1). Figure 5B shows the R_s_ values between the first and second half data (1 min 53 s per half). CSF BS resulted in the highest reproducibility (R_s_ = 0.89 ± 0.05) when compared to regular BS (R_s_ = 0.57 ± 0.17, *p* < 0.001) and enhanced BS (0.64 ± 0.15, *p* < 0.01).

**FIGURE 6:**
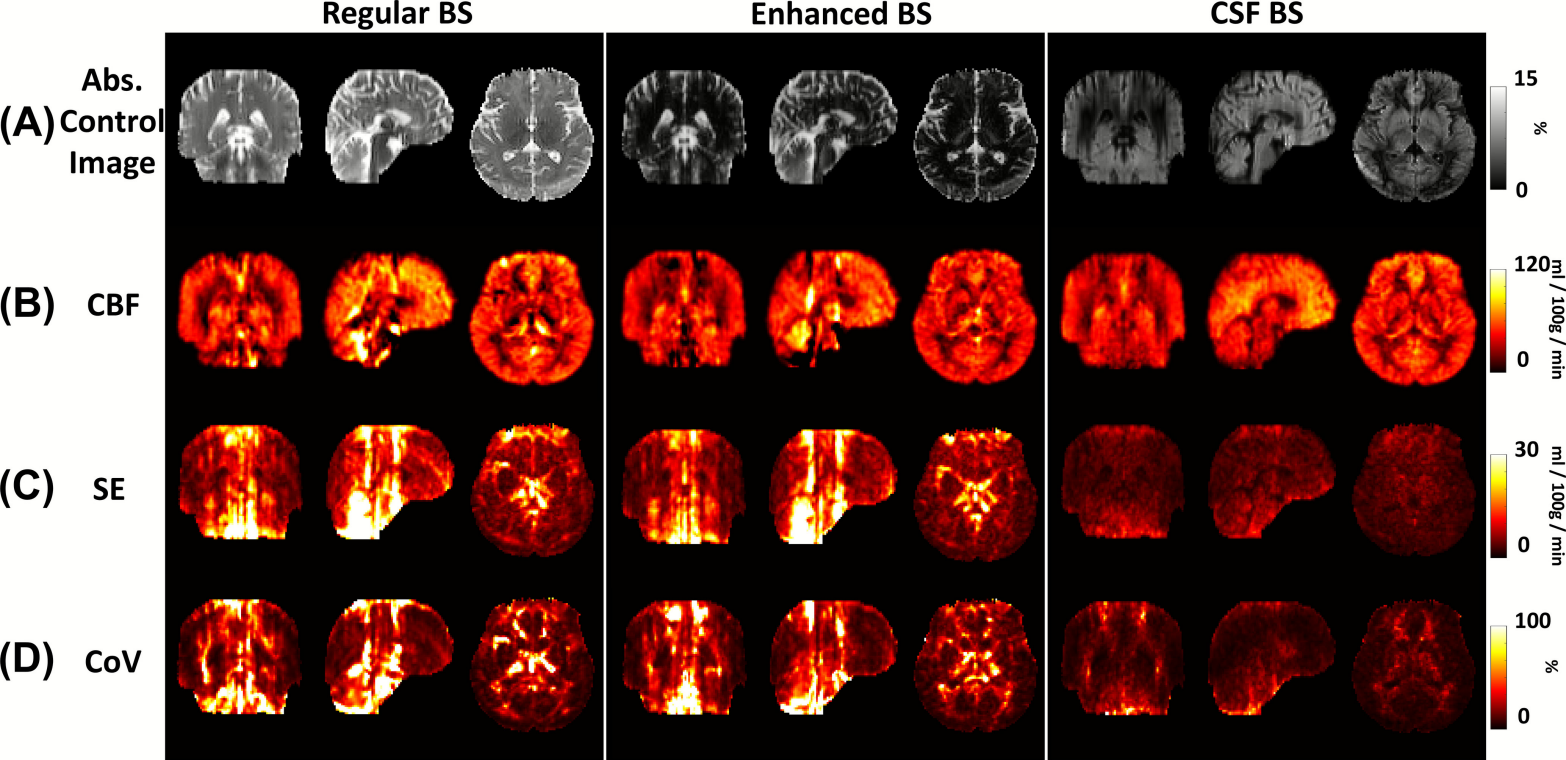
Images of different BS schemes using segmented 3D GRASE acquisition in a representative subject. (A) Absolute control image. (B) Cerebral blood flow (CBF) maps acquired with different BS schemes. (C) Temporal standard error (SE) maps across measurements. (D) Coefficient‐of‐variation (CoV) maps.

Similar results were found in the segmented acquisition. Figure 6 shows representative segmented pCASL data using different BS schemes. Figure 6A shows the corresponding control images. The artifacts remained evident in the regular and enhanced BS CBF images (Figure 6B), with high SE and CoV values in regions rich in pulsatile CSF (Figure 6C,D). CSF BS effectively suppressed artifacts in the CBF maps (Figure 6B) and reduced signal fluctuations (Figure 6C,D). Whole‐brain CBF values were not different across BS schemes (ANOVA, *p* = 0.14). Voxel‐wise CoV distributions (Figure 5C) again demonstrated that CSF BS yielded the least outlier voxels. Mean voxel‐wise CoV values were 11.3 ± 2.5%, 24.6 ± 4.4%, and 26.7 ± 6.2% when using CSF, enhanced, and regular BS schemes, respectively. CSF BS resulted in the highest R_s_ values (0.86 ± 0.04; Figure 5D), when compared to regular BS (0.61 ± 0.12, *p* < 0.001) and enhanced BS (0.65 ± 0.10, *p* < 0.001).

### 3.2 Study 2: Comparison of BS Schemes in Multi‐Delay pCASL

**FIGURE 7:**
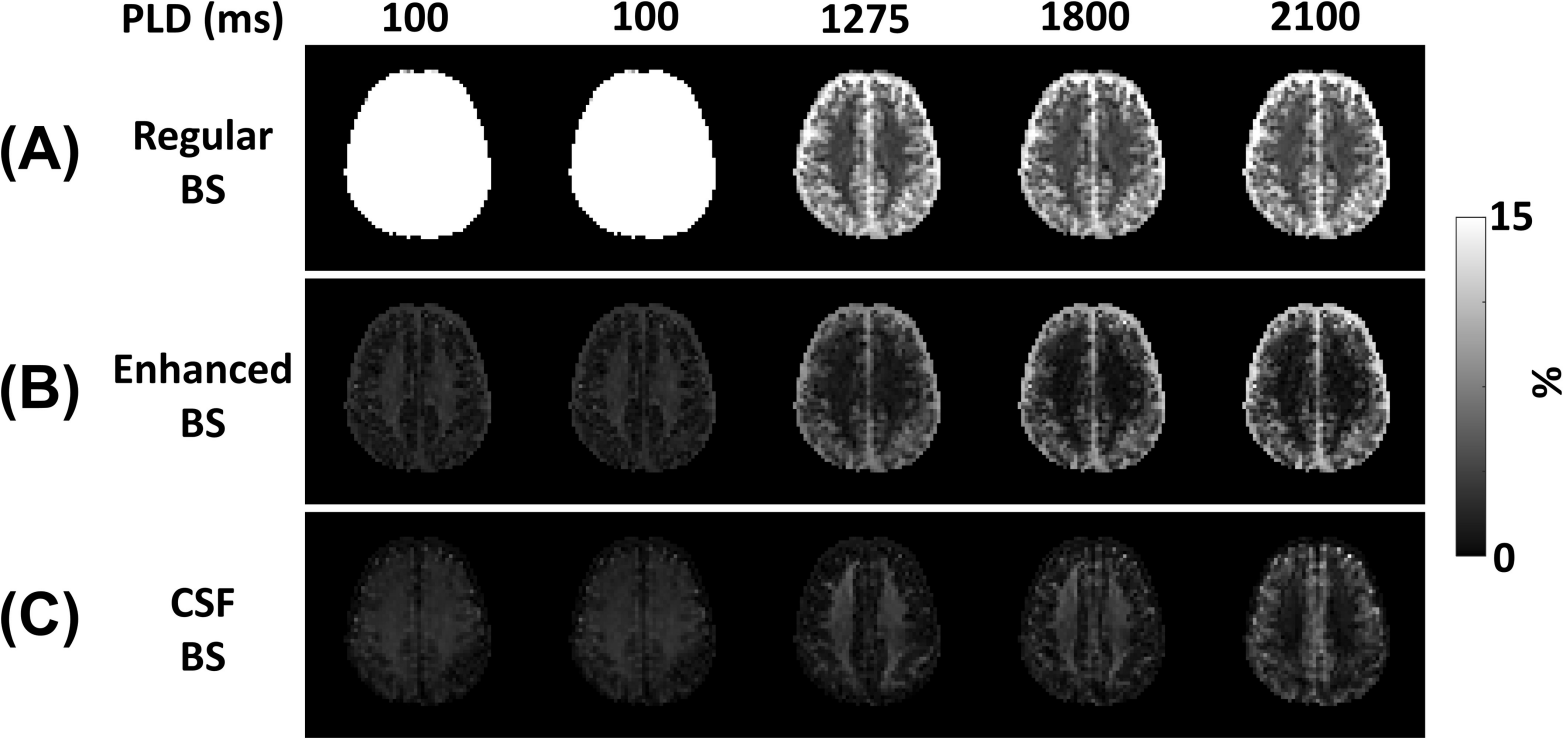
ASL control images for each PLD across different background suppression (BS) schemes. Signals are expressed as percentage of the M0 signal. (A) Regular BS. The constrained inversion pulse timing was less effective at short PLD (100 ms), resulting in high residual signals (gray and white matter > 50% and CSF > 20%). (B) Enhanced BS. Gray and white matter tissue signals were minimized across PLDs. (C) CSF BS. CSF signals were robustly attenuated at all PLDs.

Figure 7 displays representative control images for each PLD. The labeled images were visually the same; thus, they were not shown. The regular BS scheme using constrained inversion pulse timings (Figure 7A) was less effective at short PLD (100 ms), with high residual signals (gray and white matter > 50% and CSF > 20%). In contrast, the enhanced and CSF BS schemes using unconstrained inversion pulse timings (Figure 7B,C) were effective across all PLDs. Enhanced BS consistently minimized gray and white signals, while CSF BS robustly attenuated CSF signals.

**FIGURE 8:**
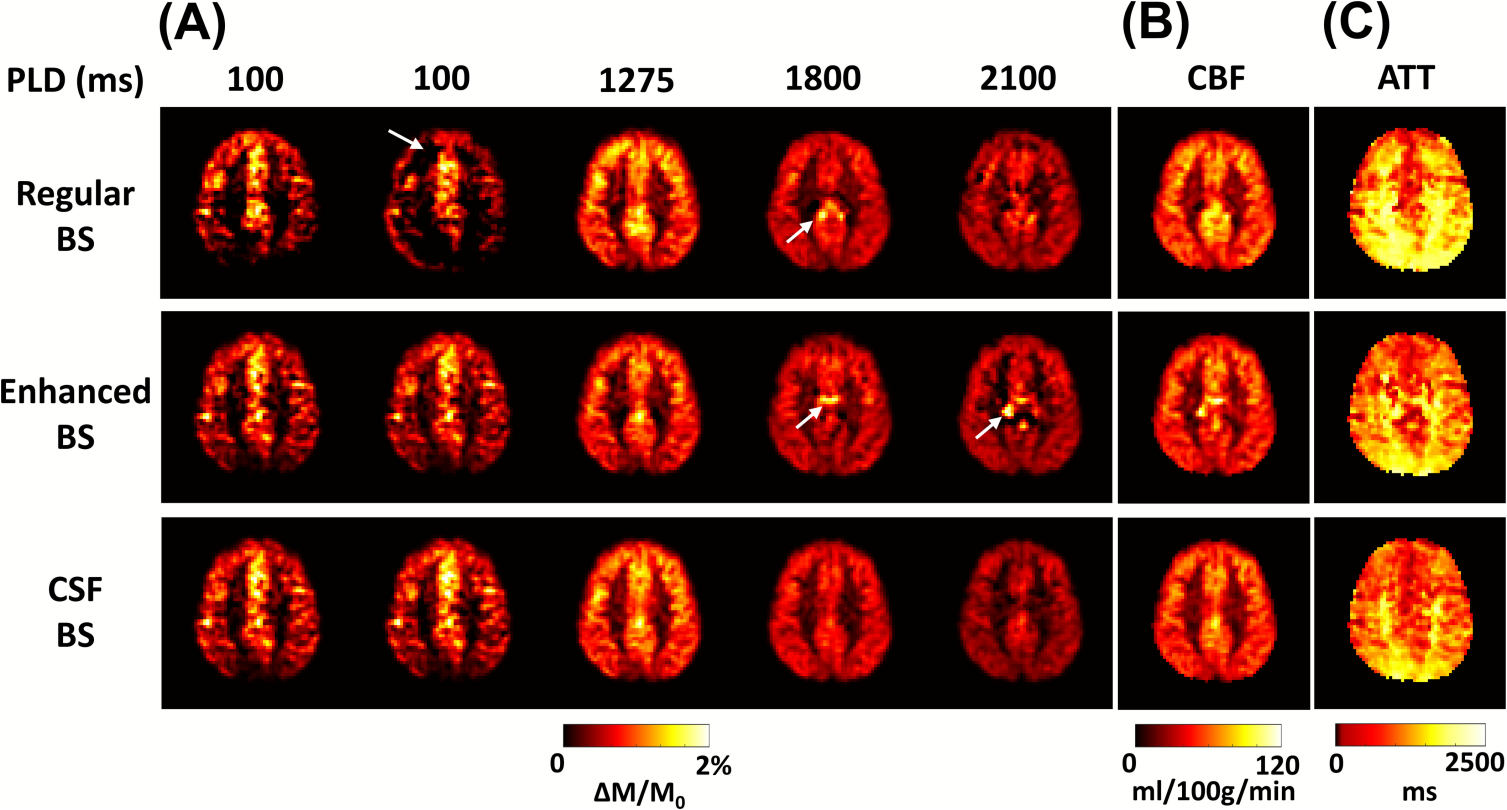
Multi‐delay pCASL results acquired with different BS schemes. (A) CBF‐weighted images acquired at five PLDs. The signals have been scaled by M0. White arrows indicate examples of spurious signals presented in the regular and enhanced BS schemes. (B) Fitted CBF maps. (C) Fitted ATT maps.

Figure 8 shows CBF‐weighted images as a function of PLD, along with the corresponding CBF and ATT maps derived from fitting. Visual inspection suggested that CSF BS produced homogeneous images across all PLDs (Figure 8A). In contrast, the regular and enhanced BS schemes revealed some spurious signals (white arrows). Consequently, the quality of CBF (Figure 8B) and ATT (Figure 8C) maps was higher in the CSF BS scheme compared to the other BS schemes.

**FIGURE 9:**
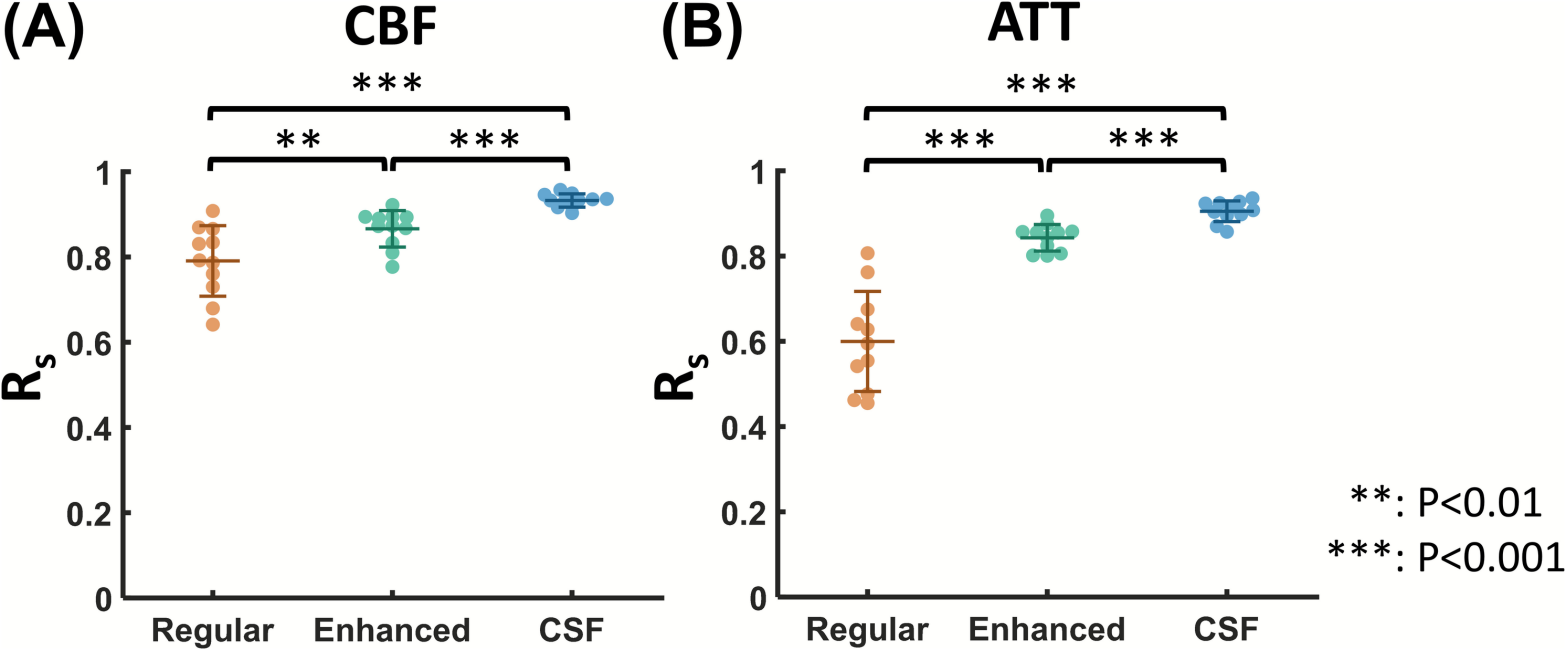
Reproducibility of CBF and ATT measured with multi‐delay ASL. (A) Spearman correlation coefficient (R_s_) values between CBF maps from two repeated runs. (B) R_s_ values between ATT maps from two repeated runs. Error bars denote the standard deviation across subjects. The *p* values were obtained using Tukey's post hoc test following repeated‐measures ANOVA.

Table 2 summarizes the voxel‐wise CoV values from the test–retest data. CSF BS consistently yielded the lowest CoV across all PLDs. For CBF maps, CSF BS showed the lowest CoV (5.9 ± 0.8%), followed by enhanced BS (8.0 ± 1.8%, *p* = 0.003 when compared to CSF BS) and regular BS (9.9 ± 2.3%, *p* < 0.001 when compared to CSF BS). Similar findings were observed for ATT, with a CoV of 11.3 ± 2.9%, 13.9 ± 1.9%, and 21.3 ± 3.2% for CSF, enhanced, and regular BS schemes, respectively. When examining the spatial R_s_ values of the test–retest data (Figure 9), CSF BS outperformed the other BS schemes by achieving the highest R_s_ of 0.93 in CBF (Figure 9A) and 0.91 in ATT (Figure 9B).

**TABLE 2:** Voxel‐wise CoV values of different BS schemes.

CoV (%)	PLD = 100 ms	PLD = 100 ms	PLD = 1275 ms	PLD = 1800 ms	PLD = 2100 ms	CBF	ATT
Regular BS	56.3 ± 20.0	57.9 ± 20.2	18.7 ± 5.0	25.9 ± 7.7	37.1 ± 10.2	9.9 ± 2.3	21.3 ± 3.2
Enhanced BS	12.0 ± 5.1	12.5 ± 5.1	14.0 ± 4.5	22.0 ± 5.8	28.2 ± 4.8	8.0 ± 1.8	13.9 ± 1.9
CSF BS	12.0 ± 4.1	12.1 ± 3.5	8.8 ± 3.4	12.8 ± 4.3	19.2 ± 7.2	5.9 ± 0.8	11.3 ± 2.9
ANOVA *p*	< 0.001	< 0.001	< 0.001	< 0.001	0.002	< 0.001	< 0.001
Regular vs. enhanced	< 0.001	< 0.001	0.002	0.19	0.008	0.009	< 0.001
Regular vs. CSF	< 0.001	< 0.001	< 0.001	< 0.001	0.002	< 0.001	< 0.001
Enhanced vs. CSF	0.94	0.48	< 0.001	< 0.001	0.01	0.003	0.01

*Note:* P values were obtained by Bonferroni‐corrected repeated measures ANOVA, followed by the Tukey post hoc test.

Abbreviations: ATT = arterial transit time, BS = background suppression, CBF = cerebral blood flow, CoV = coefficient of variation, PLD = post‐labeling delay.

## 4 Discussion

In this study, we investigated different BS schemes in ASL and found that a CSF BS scheme in combination with complex image subtraction yielded the most benefits in improving ASL data quality. Compared to gray and white matter focused BS, CSF BS effectively reduced artifacts caused by fluid pulsation, which could plaque brain regions beyond CSF‐rich areas. Compared to regular BS, CSF BS reduced CoV of perfusion signal by nearly 50%. With the proposed CSF BS scheme, a multi‐delay pCASL scan of 5 min can yield CBF and ATT maps with an R_s_ of 0.93 and 0.91, respectively.

Noise in ASL MRI can be attributed to thermal and, more importantly, physiological origins. Physiological noise is proportional to the amount of static tissue signal, which underscores the importance of BS. This presents a cost‐effective opportunity to improve the quality of ASL MRI data [14, 15], in particular because the signal in ASL is often limited by physical boundaries such as the number of spins reaching the tissue and the T1 decay of the labeling effect [33]. To date, most BS schemes have focused on suppressing gray and white matter signals [16, 17, 18, 19]. CSF signals, on the other hand, have received less attention. It was previously thought that CSF fluctuations were predominantly observed in the fourth ventricles and nearby regions [34] and that CSF outside the brain does not manifest pulsatile flow. Recently surging interests in neurofluid circulation have shed new insights on the CSF flow. Reports using diffusion, phase‐contrast, and velocity‐selective MRI methods have demonstrated converging evidence that CSF in the subarachnoid space indeed exhibits strong pulsatile motion [20, 21, 22, 35]. These physiological fluctuations are expected to induce substantial noise in the ASL data. Therefore, we hypothesized that a BS scheme focusing on suppressing CSF signal may provide superior performance compared to gray/white matter suppression schemes. The results in this report appear to support our hypothesis. Pulsatile CSF introduced substantial signal instability resulting in imperfect control/label signal cancellation, the consequence of which is bright and dark spurious artifacts in CBF maps. Due to the 3D acquisition that is commonly employed in ASL, these artifacts can further extend to neighboring slices and degrade image quality even in regions not rich in CSF. This effect is exacerbated by long acquisition echo‐train, because CSF has a high T2 and its signal is more prominent at extended echo times. Therefore, by minimizing the CSF signal, the proposed CSF BS scheme effectively mitigated artifacts and improved the stability of perfusion measurements. An additional advantage of CSF BS is that it attenuates signals from the eyes, which are fluid‐filled structures with similar T_1_ relaxation values as CSF. As a result, eye‐motion‐related aliasing artifacts in segmented acquisitions are substantially reduced, allowing greater flexibility in the choice of k‐space segmentation strategies.

In the present study, the proposed CSF BS scheme provided voxel‐wise CoV values of 5.9% for CBF and 11.3% for ATT when using a 5‐min multi‐delay scan. Reproducibility of ASL has been investigated in several prior studies. Using pulsed ASL, the QUASAR (quantitative STAR labeling of arterial regions) study reported a gray matter CoV of 6%–15% [36]. The work by Chen et al. demonstrated that single‐delay pCASL yielded a gray matter CoV of 3.5% [37]. Other studies reported gray matter CoV values in the range of 5%–15% [38, 39]. In the multi‐delay setting, Guo et al. observed CoV values of 24%–28% for CBF and 29%–33% for ATT [40]. The other reproducibility study of multi‐delay ASL did not report the CoV of ATT but only calculated that of gray matter CBF, which was 4.8% [41]. The CoV values reported in this study compare favorably to those in the literature [36, 37, 38, 39, 40, 41, 42], suggesting that reliable CBF and ATT estimations can be achieved when the CSF BS scheme is used. Such benefits may be useful in clinical settings.

The concept of BS was originally proposed for MR angiogram [13]. Ye et al. were the first to propose BS in ASL MRI [14]. It was later applied to 2D and 3D ASL studies [15, 17]. These early studies used constrained BS, in that the inversion pulses could only be placed in the PLD period. Most vendors' current implementations of ASL have been based on constrained BS. Dai et al. and Maleki et al. extended it to unconstrained BS, allowing the inversion pulses to be placed during the labeling period as well [16, 43]. Guo et al. proposed projection‐based complex image subtraction to further suppress background signals [26]. The present study found that constrained BS was not able to provide sufficient suppression in short PLD protocols. This presents a limitation in multi‐delay ASL, which requires some data at short delay times. In contrast, unconstrained BS allows improved suppression and superior signal stability at short PLD timing [16]. However, it should be pointed out that unconstrained BS also has its limitations. Specifically, BS inversion pulses applied during the labeling period need to be slab selective (to spins distal to the labeling plane) in order for the labeled spins before and after the inversion pulses not to cancel each other out. With this approach, arterial spins proximal to the labeling plane will not experience the BS pulses; thus, they can appear as bright or dark spurious signals after the control and label subtraction. Additional BS strategies such as arterial saturation that suppresses the arterial spins arriving after the labeling module [17, 19, 44, 45] are needed to reduce such artifacts. Therefore, constrained and unconstrained BS each have advantages and limitations, and their application should be considered depending on specific sequence needs. As an additional note, the BS inversion pulses during the PLD period should be nonselective rather than selective to the spins distal to the labeling plane in order to reduce pulsation noise in arterial regions.

This study also conducted a comparison between regular BS and enhanced BS schemes. It was found that the enhanced scheme provided better performance than regular BS in multi‐delay pCASL, but not in the single‐delay data. This discrepancy is likely because the single‐delay scans used a long echo‐train length; thus, the gray and white matter signal is further suppressed during the acquisition.

It should be pointed out that, while the CSF BS scheme was implemented within a pCASL framework in the present study, this approach can be easily adapted to other ASL sequences, such as pulsed ASL or velocity‐selective ASL, as well as to alternative timing–encoding schemes such as Hadamard encoding. The proposed BS scheme is fully compatible with advanced acquisition strategies, such as parallel imaging and compressed sensing [46, 47], which can help reduce echo‐train length and motion sensitivity. Furthermore, it can be combined with other denoising methods, including deep learning–based techniques [48, 49] to further enhance ASL data quality.

This study has several limitations. First, the new CBF reconstruction algorithm involves projecting ASL control and label images onto the M0 image. Thus, any motion between ASL and M0 acquisitions can negatively affect the results. Motion correction performed separately on real and imaginary images can mitigate this issue. Second, while CSF BS effectively suppresses CSF signal, it does not minimize residual gray and white matter signals (3%–7% in the present study). These static tissue signals may contribute to the noise. Additional inversion pulses can be used to broaden the range of T1 suppression. However, every extra inversion pulse will result in a reduction in ASL signal due to inversion imperfection. Thus, the present study used two inversion pulses. Third, acquisitions in Study 1 used relatively long echo‐train lengths (~1 s), which will cause image blurring along the slice‐encoding direction. We estimated that the point‐spread function (PSF) for tissue under the acquisitions in Study 1 is 10.5–18.9 mm. In contrast, the PSF for CSF is considerably narrower (~2.3 mm) because CSF has a much longer T2 relaxation time (~2 s), leading to less signal decay during the readout. Thus, long readouts primarily cause blurring in tissue signals but not in CSF signals. Despite this blurring effect with long echo‐train length, we point out that we have also tested to use a standard echo‐train length (360 ms, with a tissue PSF of 5.5 mm) in Study 2, and the results were similar. Therefore, our data suggest that CSF BS provides an SNR benefit for a variety of acquisition schemes. We also note that tradeoffs between spatial resolution and SNR are often needed in ASL studies. For example, long echo‐train length and short scan duration may be imperative in studies of hyperacute stroke, ASL‐based cerebrovascular reactivity mapping, or pediatric perfusion imaging. Thus, it is useful to test the benefits of CSF BS in single‐shot acquisitions. Finally, saving raw k‐space data can become a burden on clinical workflow. This can be mitigated by making modifications to the scanner's reconstruction code so that real and imaginary images can be included in the sequence output.

## 5 Conclusion

This study proposed a CSF‐focused BS scheme for improving data quality in ASL perfusion imaging. This method effectively reduced artifacts from CSF pulsation and increased signal stability in both single‐delay and multi‐delay pCASL. This BS strategy may support the broader utilization of ASL in clinical applications.

## Author Contributions

**Z.H.:** conception and design of the study. **H.L.:** conception and design of the study. **Z.H.:** data acquisition. **W.S.:** data acquisition. **Y.G.:** data acquisition. **Z.H.:** data analysis. **Z.W.:** data analysis. **Z.H.:** data interpretation. **V.S.Y.:** data interpretation. **D.D.L.:** data interpretation. **H.L.:** data interpretation. **Z.H.:** manuscript writing. **H.L.:** manuscript writing. All authors: critical revision and approval of the manuscript.

## Funding

This work was supported by the National Institutes of Health, through R01 AG085299, R01 NS106711, R01 AG064792, R01 AG071515, R01 EB037564, U01 NS100588, R21 AG079098, and P41 EB031771.

## Conflicts of Interest

The authors declare no conflicts of interest.

## Supporting information


**Figure S1:** Group‐level voxel‐wise comparison of CoV maps under different background suppression (BS) schemes using (A) single‐shot 3D GRASE acquisition, and (B) segmented 3D GRASE acquisition. CSF BS was compared with enhanced BS, which was used as the representative tissue‐focused scheme. For each subject, CoV difference map (CoV_enhanced_ − CoV_CSF_) were computed and entered into a group‐level one‐sample *t*‐test. Color bars indicate *t*‐scores. Warm colors (red–yellow) represent voxels where CSF BS yielded lower CoV, and cool colors (blue) represent voxels where CSF BS yielded higher CoV. Statistical maps were thresholded at *p* < 0.01 and overlaid on a T1‐weighted atlas. Lower CoV with CSF BS was primarily observed in regions rich in both CSF and large arteries.
**Table S1:** Inversion time and simulated residual signals of different BS schemes optimized for each PLD.

## Data Availability

The data that support the findings of this study are available on request from the corresponding author. The data are not publicly available due to privacy or ethical restrictions.
