# Supplementary material for: A CSF Background Suppression Scheme in Arterial Spin Labeling MRI
Source: NMR Biomed. 2025 Nov 29;39(1):e70191. doi: 10.1002/nbm.70191 (PMC12664665; doi:10.1002/nbm.70191)
Supplement: Supplementary file 1 — Figure S1: Group‐level voxel‐wise comparison of CoV maps under different background suppression (BS) schemes using (A) single‐shot 3D GRASE acquisition, and (B) segmented 3D GRASE acquisition. CSF BS was compared with enhanced BS, which was used as the representative tissue‐focused scheme. For each subject, CoV difference map (CoVenhanced − CoVCSF) were computed and entered into a group‐level one‐sample t‐test. Color bars indicate t‐scores. Warm colors (red–yellow) represent voxels where CSF BS yielded lower CoV, and cool colors (blue) represent voxels where CSF BS yielded higher CoV. Statistical maps were thresholded at p < 0.01 and overlaid on a T1‐weighted atlas. Lower CoV with CSF BS was primarily observed in regions rich in both CSF and large arteries. Table S1: Inversion time and simulated residual signals of different BS schemes optimized for each PLD. [file NBM-39-e70191-s001.docx]

**
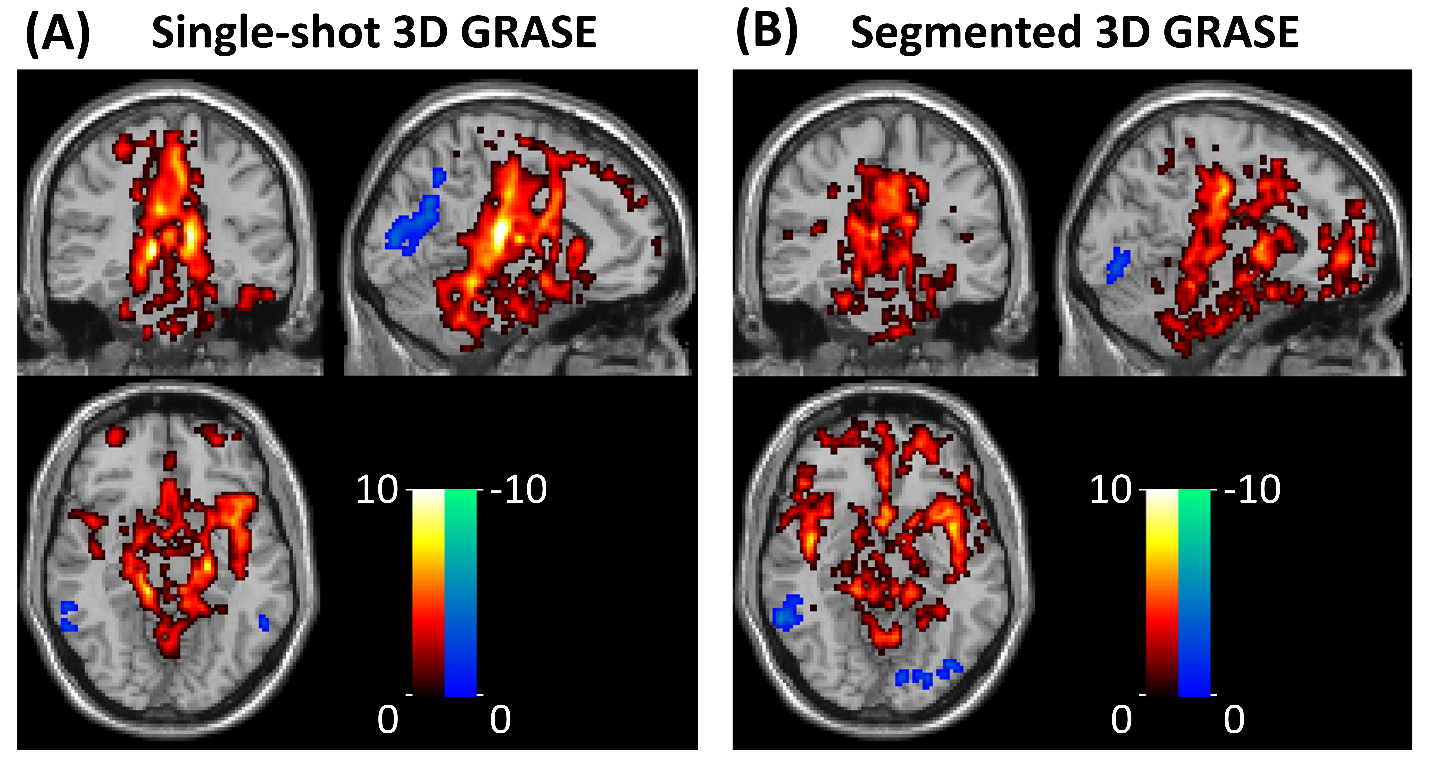
**

**Supporting Information Supplemental Figure S1.** Group-level voxel-wise comparison of CoV maps under different background suppression (BS) schemes using (A) single-shot 3D GRASE acquisition, and (B) segmented 3D GRASE acquisition. CSF BS was compared with enhanced BS, which was used as the representative tissue-focused scheme. For each subject, CoV difference map (CoV_enhanced_ - CoV_CSF_) were computed and entered into a group-level one-sample t-test. Color bars indicate t-scores. Warm colors (red–yellow) represent voxels where CSF BS yielded lower CoV, and cool colors (blue) represent voxels where CSF BS yielded higher CoV. Statistical maps were thresholded at p < 0.01 and overlaid on a T1-weighted atlas. Lower CoV with CSF BS was primarily observed in regions rich in both CSF and large arteries.

**Supporting Information Supplemental Table S1.** Inversion time and simulated residual signals of different BS schemes optimized for each PLD

| **BS scheme** | **PLD (ms)** | **TI_BS1_ (ms)** | **TI_BS2_ (ms)** | **GM (%)** | **WM (%)** | **CSF (%)** |
| --- | --- | --- | --- | --- | --- | --- |
| Regular | 100 | 1806 | 1874 | 59.2 | 65.0 | 28.1 |
|  | 100 | 1806 | 1874 | 59.2 | 65.0 | 28.1 |
|  | 1275 | 1806 | 2767 | 6.4 | 3.6 | 11.6 |
|  | 1800 | 2096 | 3235 | 4.6 | 4.7 | 11.8 |
|  | 2100 | 2346 | 3525 | 4.7 | 4.9 | 13.2 |
|  |  |  |  |  |  |  |
| Enhanced | 100 | 796 | 1627 | 0.05 | 0.1 | 2.1 |
|  | 100 | 796 | 1627 | 0.05 | 0.1 | 2.1 |
|  | 1275 | 1616 | 2744 | 0.1 | -0.02 | 6.8 |
|  | 1800 | 2026 | 3248 | 0.2 | 0.5 | 9.2 |
|  | 2100 | 2296 | 3546 | 0.2 | 0.1 | 10.9 |
|  |  |  |  |  |  |  |
| CSF | 100 | 666 | 1580 | -1.4 | 1.6 | -0.06 |
|  | 100 | 666 | 1580 | -1.4 | 1.6 | -0.06 |
|  | 1275 | 1236 | 2640 | -2.3 | 5.6 | 0.3 |
|  | 1800 | 1523 | 3132 | -3.3 | 6.8 | 0.2 |
|  | 2100 | 1734 | 3445 | -6.0 | 3.9 | -0.08 |

**Abbreviations:** BS = background suppression, TI = inversion time after pre-saturation, GM = gray matter, WM = white matter.
